# *rac*-(*E*)-3-[1-(2-Chloro­phen­yl)eth­yl]-5-methyl-*N*-nitro-1,3,5-oxadiazinan-4-imine

**DOI:** 10.1107/S1600536810034343

**Published:** 2010-08-28

**Authors:** Cong-Cong Li, Yuan-Yuan Zhong, Liang-Zhong Xu

**Affiliations:** a College of Chemistry and Molecular Engineering, Qingdao University of Science and Technology, Qingdao 266042, People’s Republic of China

## Abstract

In the title compound, C_12_H_15_ClN_4_O_3_, which has potential insecticidal activity, the oxadiazine ring and the benzene ring make a dihedral angle of 84.63 (2)° to one another. The crystal packing involves weak inter­molecular C—H⋯O hydrogen bonds.

## Related literature

For the biological activity of oxadiazine derivatives, see: Maienfisch & Huerlimann (1994 ▶); Gsell & Maienfisch (1998 ▶). For the synthesis, see: Gottfied *et al.* (2001 ▶). For related structures, see: Chopra *et al.* (2004 ▶); Kang *et al.* (2008 ▶); Zhong *et al.* (2010 ▶). For puckering parameters, see: Cremer & Pople (1975 ▶).
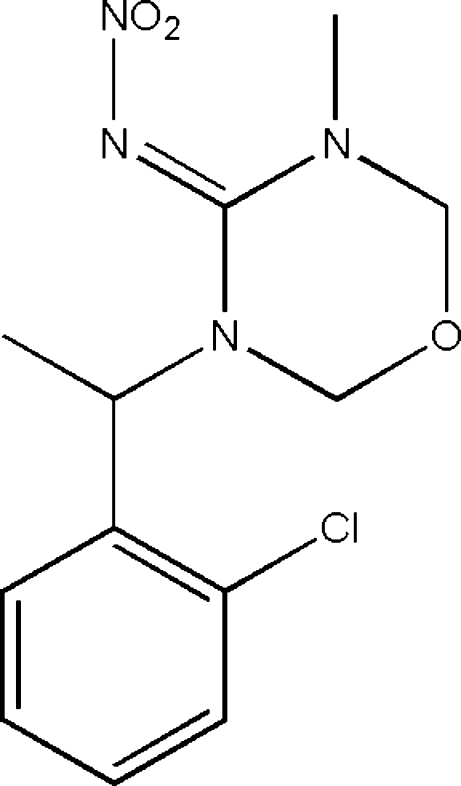


## Experimental

### 

#### Crystal data

C_12_H_15_ClN_4_O_3_
*M* *_r_* = 298.73
Monoclinic, 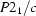

*a* = 17.259 (4) Å
*b* = 6.9157 (14) Å
*c* = 12.169 (2) Å
β = 109.63 (3)°
*V* = 1368.0 (5) Å^3^
*Z* = 4
Cu *K*α radiation
μ = 2.61 mm^−1^
*T* = 113 K
0.26 × 0.22 × 0.18 mm

#### Data collection

Rigaku Saturn diffractometer
Absorption correction: multi-scan (*CrystalClear*; Rigaku, 2005 ▶) *T*_min_ = 0.550, *T*_max_ = 0.651
12087 measured reflections
2590 independent reflections
2562 reflections with *I* > 2σ(*I*)
*R*_int_ = 0.054

#### Refinement

*R*[*F* ^2^ > 2σ(*F* ^2^)] = 0.039
*wR*(*F* ^2^) = 0.104
*S* = 1.08
2590 reflections
184 parameters
H-atom parameters constrained
Δρ_max_ = 0.26 e Å^−3^
Δρ_min_ = −0.37 e Å^−3^

### 

Data collection: *CrystalClear* (Rigaku, 2005 ▶); cell refinement: *CrystalClear*; data reduction: *CrystalClear*; program(s) used to solve structure: *SHELXS97* (Sheldrick, 2008 ▶); program(s) used to refine structure: *SHELXL97* (Sheldrick, 2008 ▶); molecular graphics: *ORTEPIII* (Burnett & Johnson, 1996 ▶); software used to prepare material for publication: *SHELXTL* (Sheldrick, 2008 ▶).

## Supplementary Material

Crystal structure: contains datablocks I, global. DOI: 10.1107/S1600536810034343/zs2061sup1.cif

Structure factors: contains datablocks I. DOI: 10.1107/S1600536810034343/zs2061Isup2.hkl

Additional supplementary materials:  crystallographic information; 3D view; checkCIF report

## Figures and Tables

**Table 1:** Hydrogen-bond geometry (Å, °)

*D*—H⋯*A*	*D*—H	H⋯*A*	*D*⋯*A*	*D*—H⋯*A*
C1—H1*A*⋯O2^i^	0.99	2.52	3.2817 (18)	134
C1—H1*A*⋯O3^i^	0.99	2.50	3.396 (2)	150
C1—H1*B*⋯O2^ii^	0.99	2.56	3.2555 (18)	127
C3—H3*A*⋯O3^iii^	0.99	2.49	3.2294 (19)	131
C4—H4*B*⋯O2^i^	0.98	2.57	3.3070 (19)	132
C4—H4*C*⋯O1^ii^	0.98	2.49	3.377 (2)	150
C6—H6*C*⋯O3^iii^	0.98	2.35	3.3020 (19)	164

Symmetry codes: (i) 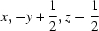; (ii) 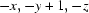; (iii) 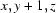.

**Fig. 1.**
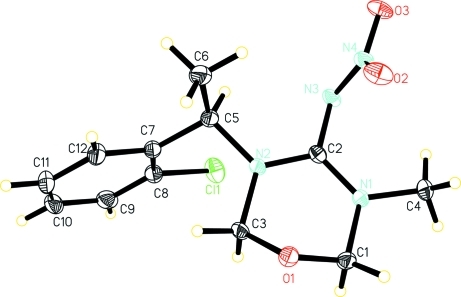
Molecular configuration and atom numbering scheme for the title compound (I), with displacement ellipsoids drawn at the 50% probability level.

## supplementary crystallographic information

### Comment

Currently, studies on oxadiazine derivatives have mainly concentrated on compounds with oxadiazine as the only active group (Gsell, *et al.*, 1998) and a number of highly insecticidal compounds of this type have been synthesized (Maienfisch *et al.*, 1994). We report here the synthesis and crystal structure of the title compound C_12_H_15_Cl_1_N_4_O_3_ (I).

In (I) (Fig. 1) the bond lengths and angles of the oxadiazine rings are in agreement with those in previous reported structures (Chopra *et al.*, 2004). The 1,3,5-oxadiazinane ring is in a half-chair conformation and the ring-puckering parameters (Cremer & Pople, 1975;) were calculated as Q = 0.05126 (12) Å; θ = 121.33 (13)°; φ = 166.3676 (15)°. The N3═O bondlength [1.3904 (17) Å] is close to the value reported in the literature (Zhong *et al.*, 2010). The oxadiazine ring and the benzene ring make a dihedral angle of 84.63 (2)°. Weak intermolecular C—H···O hydrogen bonds give a three-dimensional network (Table 1).

### Experimental

A solution of 1-(1-bromoethyl)-2-chlorobenzene (4.3 g, 20 mmol), *N*-nitro-1,3,5-oxadiazinan-4-imine (3.2 g, 20 mmol) and potassium carbonate (2.8 g, 20 mmol) in 20 g of acetonitrile was heated under reflux for 4 h. Upon cooling to room temperature the solution was filtered and then concentrated under reduced pressure to give the title compound (I) (7.89 g, 90% yield) (Gottfried, *et al.*, 2001). Single crystals suitable for X-ray measurement were obtained by recrystallization from ethanol at room temperature.

### Refinement

All C-bound H atoms were placed in calculated positions, with C—H = 0.95-0.97 Å, and included in the final cycles of refinement using a riding model, with *U*_iso_(H) = 1.2*U*_eq_(C).

### Crystal data

**Table:**

C_12_H_15_ClN_4_O_3_	*F*(000) = 624
*M**_r_* = 298.73	*D*_x_ = 1.450 Mg m^−^^3^
Monoclinic, *P*2_1_/*c*	Cu *K*α radiation, λ = 1.54187 Å
Hall symbol: -P 2ybc	Cell parameters from 1058 reflections
*a* = 17.259 (4) Å	θ = 27.5–71.9°
*b* = 6.9157 (14) Å	µ = 2.61 mm^−^^1^
*c* = 12.169 (2) Å	*T* = 113 K
β = 109.63 (3)°	Block, colorless
*V* = 1368.0 (5) Å^3^	0.26 × 0.22 × 0.18 mm
*Z* = 4	

### Data collection

**Table:**

Rigaku Saturn diffractometer	2590 independent reflections
Radiation source: fine-focus sealed tube	2562 reflections with *I* > 2σ(*I*)
graphite	*R*_int_ = 0.054
Detector resolution: 14.63 pixels mm^-1^	θ_max_ = 72.1°, θ_min_ = 2.7°
ω scans	*h* = −20→21
Absorption correction: multi-scan (*CrystalClear*; Rigaku, 2005)	*k* = −8→8
*T*_min_ = 0.550, *T*_max_ = 0.651	*l* = −13→14
12087 measured reflections	

### Refinement

**Table:**

Refinement on *F*^2^	Secondary atom site location: difference Fourier map
Least-squares matrix: full	Hydrogen site location: inferred from neighbouring sites
*R*[*F*^2^ > 2σ(*F*^2^)] = 0.039	H-atom parameters constrained
*wR*(*F*^2^) = 0.104	*w* = 1/[σ^2^(*F*_o_^2^) + (0.058*P*)^2^ + 0.6637*P*] where *P* = (*F*_o_^2^ + 2*F*_c_^2^)/3
*S* = 1.08	(Δ/σ)_max_ = 0.001
2590 reflections	Δρ_max_ = 0.26 e Å^−^^3^
184 parameters	Δρ_min_ = −0.37 e Å^−^^3^
0 restraints	Extinction correction: *SHELXL97* (Sheldrick, 2008), Fc^*^=kFc[1+0.001xFc^2^λ^3^/sin(2θ)]^-1/4^
Primary atom site location: structure-invariant direct methods	Extinction coefficient: 0.0057 (11)

### Special details

**Table:**

Geometry. All e.s.d.'s (except the e.s.d. in the dihedral angle between two l.s. planes) are estimated using the full covariance matrix. The cell e.s.d.'s are taken into account individually in the estimation of e.s.d.'s in distances, angles and torsion angles; correlations between e.s.d.'s in cell parameters are only used when they are defined by crystal symmetry. An approximate (isotropic) treatment of cell e.s.d.'s is used for estimating e.s.d.'s involving l.s. planes.
Refinement. Refinement of *F*^2^ against ALL reflections. The weighted *R*-factor *wR* and goodness of fit *S* are based on *F*^2^, conventional *R*-factors *R* are based on *F*, with *F* set to zero for negative *F*^2^. The threshold expression of *F*^2^ > σ(*F*^2^) is used only for calculating *R*-factors(gt) *etc*. and is not relevant to the choice of reflections for refinement. *R*-factors based on *F*^2^ are statistically about twice as large as those based on *F*, and *R*- factors based on ALL data will be even larger.

### Fractional atomic coordinates and isotropic or equivalent isotropic displacement parameters (Å^2^)

**Table:**

	*x*	*y*	*z*	*U*_iso_*/*U*_eq_	
Cl1	0.36163 (2)	0.37729 (6)	−0.00540 (3)	0.02744 (17)	
N1	0.09670 (7)	0.33360 (16)	−0.04098 (10)	0.0152 (3)	
N2	0.21260 (7)	0.48468 (16)	0.08081 (10)	0.0146 (3)	
N3	0.19653 (7)	0.15435 (16)	0.10592 (10)	0.0165 (3)	
N4	0.15382 (7)	0.09523 (17)	0.17188 (10)	0.0172 (3)	
O1	0.09734 (6)	0.67120 (14)	−0.01841 (9)	0.0180 (2)	
O2	0.09602 (7)	0.19372 (16)	0.18398 (9)	0.0247 (3)	
O3	0.17457 (7)	−0.06237 (15)	0.22426 (9)	0.0238 (3)	
C1	0.06227 (8)	0.5189 (2)	−0.09525 (12)	0.0171 (3)	
H1A	0.0740	0.5363	−0.1689	0.021*	
H1B	0.0018	0.5188	−0.1139	0.021*	
C2	0.16607 (8)	0.32676 (19)	0.04897 (12)	0.0138 (3)	
C3	0.18404 (8)	0.66306 (19)	0.01450 (13)	0.0172 (3)	
H3A	0.2089	0.7767	0.0630	0.021*	
H3B	0.2007	0.6644	−0.0558	0.021*	
C4	0.04698 (9)	0.1625 (2)	−0.08861 (13)	0.0196 (3)	
H4A	0.0801	0.0459	−0.0611	0.029*	
H4B	0.0285	0.1670	−0.1740	0.029*	
H4C	−0.0010	0.1599	−0.0626	0.029*	
C5	0.28920 (8)	0.48416 (19)	0.18389 (12)	0.0171 (3)	
H5	0.3141	0.3524	0.1894	0.020*	
C6	0.26984 (10)	0.5185 (2)	0.29596 (12)	0.0225 (3)	
H6A	0.3212	0.5198	0.3627	0.034*	
H6B	0.2342	0.4147	0.3061	0.034*	
H6C	0.2418	0.6430	0.2910	0.034*	
C7	0.34952 (8)	0.6269 (2)	0.16156 (13)	0.0184 (3)	
C8	0.38573 (9)	0.5900 (2)	0.07689 (13)	0.0209 (3)	
C9	0.44068 (9)	0.7161 (2)	0.05401 (14)	0.0257 (4)	
H9	0.4640	0.6869	−0.0045	0.031*	
C10	0.46123 (10)	0.8863 (2)	0.11817 (16)	0.0305 (4)	
H10	0.4997	0.9733	0.1048	0.037*	
C11	0.42544 (10)	0.9287 (2)	0.20160 (15)	0.0308 (4)	
H11	0.4389	1.0459	0.2445	0.037*	
C12	0.37004 (9)	0.8007 (2)	0.22283 (13)	0.0247 (3)	
H12	0.3457	0.8320	0.2800	0.030*	

### Atomic displacement parameters (Å^2^)

**Table:**

	*U*^11^	*U*^22^	*U*^33^	*U*^12^	*U*^13^	*U*^23^
Cl1	0.0282 (2)	0.0206 (2)	0.0376 (3)	−0.00083 (13)	0.01641 (18)	−0.00617 (14)
N1	0.0166 (6)	0.0106 (5)	0.0167 (6)	−0.0008 (4)	0.0035 (4)	−0.0001 (4)
N2	0.0146 (5)	0.0095 (5)	0.0179 (6)	0.0011 (4)	0.0029 (4)	0.0012 (4)
N3	0.0171 (6)	0.0104 (5)	0.0226 (6)	0.0015 (4)	0.0075 (5)	0.0038 (4)
N4	0.0216 (6)	0.0110 (5)	0.0182 (6)	0.0015 (5)	0.0058 (5)	0.0019 (4)
O1	0.0167 (5)	0.0119 (5)	0.0225 (5)	0.0038 (4)	0.0030 (4)	−0.0010 (4)
O2	0.0286 (6)	0.0209 (6)	0.0301 (6)	0.0107 (4)	0.0170 (5)	0.0072 (4)
O3	0.0338 (6)	0.0117 (5)	0.0275 (6)	0.0059 (4)	0.0123 (4)	0.0083 (4)
C1	0.0168 (6)	0.0134 (6)	0.0183 (7)	0.0022 (5)	0.0021 (5)	0.0009 (5)
C2	0.0156 (6)	0.0100 (6)	0.0173 (6)	0.0016 (5)	0.0077 (5)	−0.0003 (5)
C3	0.0165 (7)	0.0099 (6)	0.0226 (7)	0.0010 (5)	0.0032 (5)	0.0035 (5)
C4	0.0204 (7)	0.0144 (6)	0.0222 (7)	−0.0049 (5)	0.0048 (6)	−0.0024 (5)
C5	0.0163 (6)	0.0123 (6)	0.0190 (7)	0.0019 (5)	0.0011 (5)	0.0014 (5)
C6	0.0274 (7)	0.0183 (7)	0.0192 (7)	0.0004 (6)	0.0042 (6)	0.0019 (5)
C7	0.0149 (6)	0.0136 (7)	0.0216 (7)	0.0010 (5)	−0.0006 (5)	0.0026 (5)
C8	0.0157 (6)	0.0157 (7)	0.0270 (8)	0.0018 (5)	0.0015 (6)	0.0030 (6)
C9	0.0182 (7)	0.0267 (8)	0.0298 (8)	0.0013 (6)	0.0047 (6)	0.0093 (6)
C10	0.0211 (8)	0.0248 (8)	0.0372 (9)	−0.0083 (6)	−0.0013 (7)	0.0108 (7)
C11	0.0302 (8)	0.0187 (7)	0.0325 (9)	−0.0086 (7)	−0.0042 (7)	0.0003 (6)
C12	0.0249 (7)	0.0185 (7)	0.0242 (7)	−0.0029 (6)	−0.0004 (6)	−0.0013 (6)

### Geometric parameters (Å, °)

**Table:**

Cl1—C8	1.7492 (16)	C4—H4B	0.9800
N1—C2	1.324 (2)	C4—H4C	0.9800
N1—C4	1.4621 (17)	C5—C7	1.524 (2)
N1—C1	1.4721 (17)	C5—C6	1.528 (2)
N2—C2	1.3336 (18)	C5—H5	1.0000
N2—C3	1.4658 (16)	C6—H6A	0.9800
N2—C5	1.4856 (17)	C6—H6B	0.9800
N3—N4	1.3237 (17)	C6—H6C	0.9800
N3—C2	1.3904 (17)	C7—C12	1.396 (2)
N4—O3	1.2526 (15)	C7—C8	1.396 (2)
N4—O2	1.2567 (16)	C8—C9	1.384 (2)
O1—C1	1.4037 (17)	C9—C10	1.391 (2)
O1—C3	1.4143 (18)	C9—H9	0.9500
C1—H1A	0.9900	C10—C11	1.386 (3)
C1—H1B	0.9900	C10—H10	0.9500
C3—H3A	0.9900	C11—C12	1.389 (2)
C3—H3B	0.9900	C11—H11	0.9500
C4—H4A	0.9800	C12—H12	0.9500
			
C2—N1—C4	123.09 (11)	H4B—C4—H4C	109.5
C2—N1—C1	121.23 (11)	N2—C5—C7	108.38 (11)
C4—N1—C1	115.58 (11)	N2—C5—C6	110.73 (11)
C2—N2—C3	118.10 (11)	C7—C5—C6	115.21 (12)
C2—N2—C5	121.43 (11)	N2—C5—H5	107.4
C3—N2—C5	120.40 (11)	C7—C5—H5	107.4
N4—N3—C2	111.90 (11)	C6—C5—H5	107.4
O3—N4—O2	120.96 (12)	C5—C6—H6A	109.5
O3—N4—N3	117.07 (11)	C5—C6—H6B	109.5
O2—N4—N3	121.95 (11)	H6A—C6—H6B	109.5
C1—O1—C3	109.70 (10)	C5—C6—H6C	109.5
O1—C1—N1	109.62 (11)	H6A—C6—H6C	109.5
O1—C1—H1A	109.7	H6B—C6—H6C	109.5
N1—C1—H1A	109.7	C12—C7—C8	117.08 (14)
O1—C1—H1B	109.7	C12—C7—C5	121.90 (14)
N1—C1—H1B	109.7	C8—C7—C5	121.02 (13)
H1A—C1—H1B	108.2	C9—C8—C7	122.70 (14)
N1—C2—N2	119.99 (12)	C9—C8—Cl1	117.52 (13)
N1—C2—N3	121.87 (12)	C7—C8—Cl1	119.78 (11)
N2—C2—N3	117.88 (12)	C8—C9—C10	118.89 (16)
O1—C3—N2	108.51 (11)	C8—C9—H9	120.6
O1—C3—H3A	110.0	C10—C9—H9	120.6
N2—C3—H3A	110.0	C11—C10—C9	119.87 (15)
O1—C3—H3B	110.0	C11—C10—H10	120.1
N2—C3—H3B	110.0	C9—C10—H10	120.1
H3A—C3—H3B	108.4	C10—C11—C12	120.38 (15)
N1—C4—H4A	109.5	C10—C11—H11	119.8
N1—C4—H4B	109.5	C12—C11—H11	119.8
H4A—C4—H4B	109.5	C11—C12—C7	121.07 (16)
N1—C4—H4C	109.5	C11—C12—H12	119.5
H4A—C4—H4C	109.5	C7—C12—H12	119.5
			
C2—N3—N4—O3	177.78 (11)	C3—N2—C5—C7	−33.89 (16)
C2—N3—N4—O2	−3.68 (18)	C2—N2—C5—C6	−83.61 (15)
C3—O1—C1—N1	55.36 (14)	C3—N2—C5—C6	93.38 (14)
C2—N1—C1—O1	−18.08 (18)	N2—C5—C7—C12	110.06 (14)
C4—N1—C1—O1	158.55 (12)	C6—C5—C7—C12	−14.60 (19)
C4—N1—C2—N2	173.37 (12)	N2—C5—C7—C8	−68.88 (16)
C1—N1—C2—N2	−10.3 (2)	C6—C5—C7—C8	166.46 (13)
C4—N1—C2—N3	−0.6 (2)	C12—C7—C8—C9	0.9 (2)
C1—N1—C2—N3	175.73 (12)	C5—C7—C8—C9	179.93 (13)
C3—N2—C2—N1	0.66 (19)	C12—C7—C8—Cl1	−178.83 (10)
C5—N2—C2—N1	177.72 (12)	C5—C7—C8—Cl1	0.15 (18)
C3—N2—C2—N3	174.91 (11)	C7—C8—C9—C10	0.4 (2)
C5—N2—C2—N3	−8.03 (19)	Cl1—C8—C9—C10	−179.86 (11)
N4—N3—C2—N1	−73.97 (16)	C8—C9—C10—C11	−1.3 (2)
N4—N3—C2—N2	111.90 (14)	C9—C10—C11—C12	1.0 (2)
C1—O1—C3—N2	−64.67 (14)	C10—C11—C12—C7	0.4 (2)
C2—N2—C3—O1	36.13 (16)	C8—C7—C12—C11	−1.3 (2)
C5—N2—C3—O1	−140.96 (12)	C5—C7—C12—C11	179.72 (13)
C2—N2—C5—C7	149.11 (13)		

### Hydrogen-bond geometry (Å, °)

**Table:**

*D*—H···*A*	*D*—H	H···*A*	*D*···*A*	*D*—H···*A*
C1—H1A···O2^i^	0.99	2.52	3.2817 (18)	134.
C1—H1A···O3^i^	0.99	2.50	3.396 (2)	150.
C1—H1B···O2^ii^	0.99	2.56	3.2555 (18)	127.
C3—H3A···O3^iii^	0.99	2.49	3.2294 (19)	131.
C4—H4B···O2^i^	0.98	2.57	3.3070 (19)	132.
C4—H4C···O1^ii^	0.98	2.49	3.377 (2)	150.
C6—H6C···O3^iii^	0.98	2.35	3.3020 (19)	164.

Symmetry codes: (i) *x*, −*y*+1/2, *z*−1/2; (ii) −*x*, −*y*+1, −*z*; (iii) *x*, *y*+1, *z*.
